# Development and validation of the Rapid Positive Mental Health Instrument (R-PMHI) for measuring mental health outcomes in the population

**DOI:** 10.1186/s12889-020-08569-w

**Published:** 2020-04-10

**Authors:** Janhavi Ajit Vaingankar, Edimansyah Abdin, Robertus Martinus van Dam, Siow Ann Chong, Linda Wei Lin Tan, Rajeswari Sambasivam, Esmond Seow, Boon Yiang Chua, Hwee Lin Wee, Wei Yen Lim, Mythily Subramaniam

**Affiliations:** 1 Research Division, Institute of Mental Health, 10, Buangkok View, Singapore, 539747 Singapore; 2 Department of Medicine, Saw Swee Hock School of Public Health, Yong Loo Lin School of Medicine, National University of Singapore and National University Health System, Singapore, Singapore; 3 Department of Clinical Epidemiology, Tan Tock Seng Hospital, Singapore, Singapore; 4 Lee Kong Chian School of Medicine, Nanyang Technological University, 50 Nanyang Avenue, Singapore, 639798 Singapore

**Keywords:** Partial Credit Rasch model, Split-half sample, Scale agreement, Multi-ethnic

## Abstract

**Background:**

The multidimensional Positive Mental Health Instrument (PMHI) has 47 items and six subscales. This study aimed to develop and validate a short unidimensional version of the PMHI among Singapore’s adult resident population.

**Methods:**

Using pooled data from three earlier studies (*n* = 1050), PMHI items were reduced by Partial Credit Rasch Model (PCRM) runs in a random split-half sample, while psychometric properties of the resulting measure were tested through confirmatory factor analysis (CFA), item response theory-graded response model and internal consistency reliability in the other half. Its reliability, construct and concurrent validity, agreement with the original scale, floor and ceiling effect, and scale estimates were further investigated in an external representative general population sample (*n* = 1925).

**Results:**

The average age of the participants was around 41 years. Four PCRM re-runs for item selection resulted in a 6-item unidimensional Rapid PMHI (R-PMHI). CFA confirmed the unidimensional structure of the R-PMHI in the internal (RMSEA = 0.075, CFI = 0.985, TLI = 0.974) and external (RMSEA = 0.051, CFI = 0.992, TLI = 0.987) validation samples. In the external validation sample, the R-PMHI met concurrent validity criteria, showing high agreement with the 47-item version with intra-class correlation coefficient of 0.872 (95% CI: 0.861 to 0.882) and low floor and ceiling effects. Weight-adjusted mean (SE, 95% CI) R-PMHI score in the population was 4.86 (0.2, 4.82–4.90).

**Conclusion:**

The unidimensional 6-item R-PMHI offers brevity over the original multidimensional measure while appropriately representing the positive mental health construct. Prospective studies are needed to assess its responsiveness and test-retest reliability.

## Background

Mental health is essential for individuals’ overall well-being and relates to their emotional, psychological and social functioning [1]. The World Health Organisation (WHO) defines mental health as a state of well-being beyond the mere absence of disease and emphasized the need for promoting mental well-being, creating space for health-oriented approaches to mental health over illness-oriented services. Several successful research and scientific efforts have since enriched our knowledge about mental health and resulted in the development of a wide array of measurement tools to assess different aspects of mental well-being and evaluate the impact of mental health interventions. The Positive and Negative Affect Scale [2], Satisfaction With Life Scale [3], Psychological Well-being Scales [4], Affectometer [5], Short Form (SF)-36 [6], WHO-Five Well-being Scale [7], Warwick Edinburgh Mental Well-being Scale (WEMWBS) [8], Positive Mental Health Instrument (PMHI) [9] and the Positive Mental Health Questionnaire [10] are some of the measures used globally in the assessment of mental well-being.

Of these, the PMHI has been widely used in Singapore's population and validated among the general population samples and mental health service users [9, 11, 12]. The multidimensional scale has 47 items representing six domains of positive mental health - general coping, emotional support, spirituality, interpersonal skills, personal growth and autonomy, and global affect. The body of work around the PMHI has so far provided important information on sociodemographic variations in the level of positive mental health in terms of gender, ethnicity and marital status in the Singapore population [13]. In general, women scored higher on emotional support and lower on personal growth and autonomy domains, non-Chinese ethnicities had higher levels for all positive mental health domains and being married (vs being separated/ divorced) was associated with higher spirituality. Studies in clinical populations have found gender differences in positive mental health of patients with schizophrenia [14] and positive association of positive mental health with life satisfaction and general functioning, and inverse relation with depressive symptom severity among patients with mental disorders [15]. A study conducted among mental health professionals found that employee positive mental health was associated with their life satisfaction and profession - compared to allied health employees, psychiatrists had lower scores on spirituality and nurses had higher scores on personal growth and autonomy [16]. More recently, population norms were estimated in a representative national sample in Singapore [12]. Current studies with the PMHI involve associations with negative aspects of mental health, lifestyle and behavioral factors (Unpublished). These findings have important practice and policy implications for mental health promotion in the general population, clinical settings and workplace.

An important limitation of the PMHI is that although it is relatively quick to administer, it can still impose considerable respondent burden in studies involving multiple assessments or in frail populations. In addition, the PMHI has a multidimensional structure and items belonging to four of the subscales are not presented in a sequential order, instead they are spread across the tool. The subscale score calculations thus involve using an algorithm making it challenging for quick, routine use in clinical practice. A short 19-item multidimensional version was previously developed in Singapore that provided advantages by halving the administration time and showing low ceiling effect [17]. However, it still does not adequately address the PMHI limitations. The availability of a much shorter unidimensional version would tackle these limitations more efficiently and enhance its applications in routine use.

This study aimed to: (i) develop a short unidimensional scale that could offer brevity over the 47-item PMHI while retaining its construct properties using a Rasch model approach on pooled data from three earlier studies, (ii) establish its reliability and internal validity in the cross validation sample, and (iii) confirm its psychometric properties by external validation in a large representative general population sample.

## Methods

Data collected in four previous cross-sectional studies were employed for this analysis. The study details are explained in other articles [9, 11, 12, 18]. The outlines of these studies are presented in Table 1 and briefly described below. Ethical approvals were obtained from the National Healthcare Group’s Domain Specific Review Board for all the studies and additional ethical approval was obtained from the National University of Singapore Institutional Review Board for the fourth study. Except for the first study where completing the study questionniare was treated as an implied consent, written informed consent was obtained from all participants. For participants aged below 21 years, consent was also obtained from a legally acceptable representative. Data from participants of the first three studies (*n* = 1050) were pooled together to form the ‘development and internal validation sample’. Data from the fourth study (*n* = 1925) served as the ‘external validation sample’.

**Table 1:** Details on studies used in the analyses

	Development and internal validation sample			External validation sample
	Study 1 (***n*** = 404)	Study 2 (***n*** = 360)	Study 3 (***n*** = 286)	Study 4 (***n*** = 1925)
**Brief description**	Study of PMH in the general population sample	Assessment of level of PMH in patients with psychiatric conditions	Assessment of level of PMH in informal caregivers of older adults	Representative general population survey of health and lifestyle behaviours
**Study period**	Dec 2010 - Feb 2011	Jan 2014 - Jun 2015	Jan 2015 - Apr 2016	Apr 2014 - Mar 2015
**Ethical aspects**	Study was approved by the Institutional Ethics Committee. Return of questionnaires was considered as implied consent.	Study was approved by the Institutional Ethics Committee. Written informed consent was obtained from all participants	Study was approved by the Institutional Ethics Committee. Written informed consent was obtained from all participants.	Study was approved by the Institutional Ethics Committee. Written informed consent was obtained from all participants and legal representatives of those aged < 21 y
**Study sample**	Singapore residents aged 21–65 years of Chinese, Malay or Indian ethnicity	Singapore residents aged 21–65 years of Chinese, Malay or Indian ethnicity	Singapore residents aged 21–65 years of Chinese, Malay or Indian ethnicity	Singapore residents aged 18–79 years of all ethnic groups
**Sampling**	Convenience, quota sampling through household visits and street intercepts	Convenience sampling through referrals from clinicians	Convenience sampling of caregivers of community-based older adults and older mental health service users	Representative general population sample, probability sampling
**Data/ Measures**	Sociodemographic information (age, gender, ethnicity, marital status, education level) PMHI (47 items)			Sociodemographic information (age, gender, ethnicity, marital status, education level) PMHI (47 items) EQ-5D-5 L, K6 distress scale
**Analyses conducted in the current study**	Random split-half sampling Item reduction using Partial Credit Rasch analysis Confirmatory factor analysis IRT-DIF by age, gender and ethnicity Internal consistency reliability			Confirmatory factor analysis Internal consistency reliability Agreement with original measure (intraclass correlation coefficient) Concurrent validity Floor and ceiling effect R-PMHI score estimates and socio-demographic correlates

### Study description

#### Study 1: Cross-sectional study of PMH in a general population sample [9]

This was a household survey conducted between December 2010 and February 2011 among 404 Singapore citizens or Permanent Residents (PRs) aged 21–65 years, of Chinese, Malay or Indian ethnicity who were literate in English langauge. Purposive quota plans were developed to ensure an equal spread by age, gender and ethnicity and by geographic locations across Singapore. One respondent was selected per household by convenience sampling. Interviewers skipped two houses, before approaching the next household. Street intercepts were also conducted at public areas such as malls, transport locations and community centres to include difficult-to-reach groups such as older PRs or English literate residents. Residents were provided a study invitation letter that explained the study and instructions to complete the self-administered questionnaire within the next 3 days using pen-and-paper personal interviews (PAPI).

#### Study 2: Patients with mental illness [11]

This study, conducted from January 2014 to June 2015, included a convenience sample of 360 psychiatric out-patients who were Singapore citizens or PRs, aged 21–65 years, belonging to Chinese, Malay or Indian ethnicity, capable of providing consent, literate in English language and having a history of schizophrenia, depression or anxiety spectrum disorders. Participants were enrolled in the study via convenience sampling by seeking referrals from healthcare professionals and self-referrals from study posters placed in the out-patient clinics. The study questionniare was interviewer-administered, however the PMHI was self-administered by the participants using PAPI.

#### Study 3: Caregivers of older adults [18]

Adult informal caregivers (*n* = 288) of older adults were enrolled in this study from a community-based cohort of caregivers who had given consent to re-contact them for future research studies and from a sample of caregivers of older mental health service users of a tertiary psychiatric hospital between January 2015 and April 2016. Participants completed the PMHI using PAPI. Two records with missing PMHI data were excluded from this analysis and a total of 286 records were included.

#### Study 4: Singapore Health 2 [12]

Singapore Health (SH) - 2 study was a nationally representative cross-sectional household survey conducted between April 2014 and March 2015 to assess the mental and physical health and lifestyle behaviors in Singapore’s general population. A sample of 1925 Singapore residents aged between 18 and 79 years residing in the west, north, north-east and south-eastern central zones in Singapore were administered the PMHI as part of the survey assessments. The PMHI was self-administered via computer-assisted personal interviewing (CAPI).

### Measures

#### Positive mental health

Positive Mental Health Instrument (PMHI) [9, 19]:The PMHI was administered in all the studies. The 47-item PMHI is a multi-dimensional measure comprising six dimensions of PMH: general coping (GC: 9 items), emotional support (ES: 7 items), spirituality (S: 7 items), interpersonal skills (IS: 9 items), personal growth and autonomy (PGA: 10 items), and global affect (GA: 5 items). The PMHI comprises positively worded items, for example, ‘I try not to let it bother me’, and ‘I try to get emotional support from family and friends’. Participants were asked to select a number showing how much the item describes them on a scale from 1 to 6, where ‘1’ represents ‘not at all like me’ and ‘6’ corresponds to ‘exactly like me’ for the first five subscales. For the ‘Global affect’ subscale, a list of five affect indicators were presented and participants were required to indicate ‘how often over the past 4 weeks they felt – calm, peaceful, etc'. using a 5-point response scale. Subscale and total PMH scores were obtained by adding scores of the respective items and dividing them by the number of items in each subscale, where higher scores indicate higher PMH.

#### Concurrent validity measures (administered in the SH-2 study)

EuroQoL-5 Dimensions-5 levels (EQ-5D-5L) [20] measures five dimensions of health related quality of life (mobility, self-care, usual activities, pain/discomfort and anxiety/depression), on a five-point rating scale from ‘no problems’ to ‘unable/extreme problems’. Responses to these five dimensions can be converted into 3125 unique EQ-5D health state descriptions ranging from 11111- no problems to 55555- disability/extreme problems on all five dimensions. EQ-5D Index scores can be derived using time trade-off values for the UK general population using cross-walk algorithm, and these scores range from − 0.594 (worse than being dead) to 1.00 (full health). It was hypothesized that the new PMHI would show significant positive correlation with EQ-5D-5L index scores. Kessler 6 Psychological Distress scale (K6) [21] contains six self-report questions about frequency of depressive and anxiety symptoms in the past 4 weeks. Participants rated these on a five-point rating scale from ‘none of the time’ to ‘all of the time’. A total score was obtained that ranged from 6 (indicating no distress) to 30 (indicating severe distress). The new PMHI was expected to show significant inverse correlation with K6 score.

#### Sociodemographic information

All four studies used structured questionnaires to obtain information on the socio-demographic background such as age, gender, ethnicity, marital status and education level of the participants.

### Psychometric and statistical analysis

#### Development and internal validation

Data from 1050 participants from the first three studies were pooled and randomly split into two samples. The first random split sample (*n* = 530) was used to develop a shortened unidimensional measure from the 47-item PMHI using Partial Credit Rasch modeling (PCRM), a Rasch model for polytomous items [22]. The aim of the item reduction was to reach a short unidimensional scale solution with less than 10 items which could offer brevity while retaining the construct. All 47 items were subjected to PCRM together regardless of their subscale affiliations. Misfitting items were dropped manually at every run if they showed a significant χ^2^ test with *p* value of < 0.05, their mean squares infit and/or outfit values exceeded the range of 0.7–1.3 and standardized infit and outfit statistics had high negative (less than − 2) or positive values (more than 2) [23–25]. The unidimensionality of the resulting scale termed as ‘Rapid PMHI (R-PMHI)’ was assessed in the other random half of the sample (*n* = 520) using confirmatory factor analysis (CFA). The CFA was conducted using polychoric item correlation matrix with the weighted least squares with mean and variance (WLSMV) adjusted chi-square statistic estimator for categorical variables. Several criteria were employed to determine the best fit model. We chose 0.3 as a cutoff for size of item loadings. Overall model fit was measured using comparative fit index (CFI), Tucker-Lewis index (TLI) and root mean square error of approximation (RMSEA) as goodness-of-fit (GOF) indices. Cutoff values above 0.95 for TLI and CFI, and values smaller than 0.08 for the RMSEA respectively were set for accepting the model fit [26, 27]. The psychometric properties of the R-PMHI were further assessed using item response theory graded response model (IRT-GRM) to estimate item difficulty and discrimination and test information function (TIF) curve. Differential item functioning (DIF) was investigated by age group (< 40, 40 and above), gender and ethnicity (Chinese, Malay, Indian). Internal consistency reliability was assessed using Cronbach’s alpha statistics.

#### External validation

CFA of the R-PMHI was repeated in an external sample from general population (*n* = 1925) to assess construct validity as above. CFA was also tested among age(< 40, 40 and above), gender (men, women) and ethnic (Chinese, Malay, Indian) groups. Internal consistency reliability was assessed. Concurrent validity of the R-PMHI was assessed by estimating concurrent validity through correlations with external measures - EQ-5D-5L Index and K6 distress scores, which were determined using Spearman’s rank correlation coefficient in the external validation sample. The level of agreement between the 6-item R-PMHI and the original 47-item PMHI version was examined using intra-class correlation coefficients (ICC) based on a two-way mixed-effect model where the individual effect was set at random and the effect of the instrument was fixed. R-PMH scores and socio-demographic correlates in the general population and floor (proportion of participants endorsing the lowest value of 1) and ceiling effects (proportion of participants endorsing the highest value of 6) were estimated.

Descriptive statistics were computed for the various samples and measures. Two-sided statistical significance was set at *p* value < 0.05. SAS (version 9.4), MPLUS, IRT-Pro and R software were used for the analyses.

## Results

### Development and internal validation

The mean (SD) age of the participants in the development and internal validation sample was 42.5 (11.9) and 41.9 (11.9) years, respectively. There were equivalent proportions of men and women with higher representation of Chinese participants (slightly above 40%) in the samples. The socio-demographic characteristics of the participants are presented in Table 2.

**Table 2:** Socio-demographic characteristics of the study samples

	Development sample (***N*** = 530)		Internal validation (***N*** = 520)		External validation Sample (***N*** = 1925)	
	Mean	SD	Mean	SD	Wt. Mean	SE
**Age (years)**	42.5	11.9	41.9	11.9	40.1	0.41
	**n**	**%**	**n**	**%**	**n**	**Wt.% (SE)**
**Age group**						
21–39 years	213	41.5	220	44.1	909	52.3 (1.4)
40 years and above	300	58.5	279	55.9	1016	47.7 (1.4)
**Gender**						
Men	266	50.3	216	41.6	921	52.1 (1.4)
Women	263	49.7	303	58.4	1004	47.9 (1.4)
**Ethnicity**						
Chinese	229	43.5	209	40.6	1149	71.1 (1.1)
Malay	139	26.4	128	24.9	320	14.1 (0.9)
Indian	158	30.0	178	34.6	366	11.0 (0.7)
Other	–	–	–	–	90	3.9 (0.5)
**Marital status**						
Never married	205	38.8	182	35.1	583	34.9 (1.4)
Married	282	53.3	288	55.5	1168	59.1 (1.4)
Divorced/ Separated/ Widowed	42	7.9	49	9.5	170	6.1 (0.6)
**Education level**						
Some/Primary	31	5.9	50	9.8	70	3.0 (0.4)
Secondary to Junior College	202	38.2	191	37.1	810	40.0 (1.4)
Vocational / Diploma	163	30.8	155	30.0	424	24.0 (1.2)
University and above	133	25.1	120	23.3	621	33.0 (1.3)

**Fig. 1:**
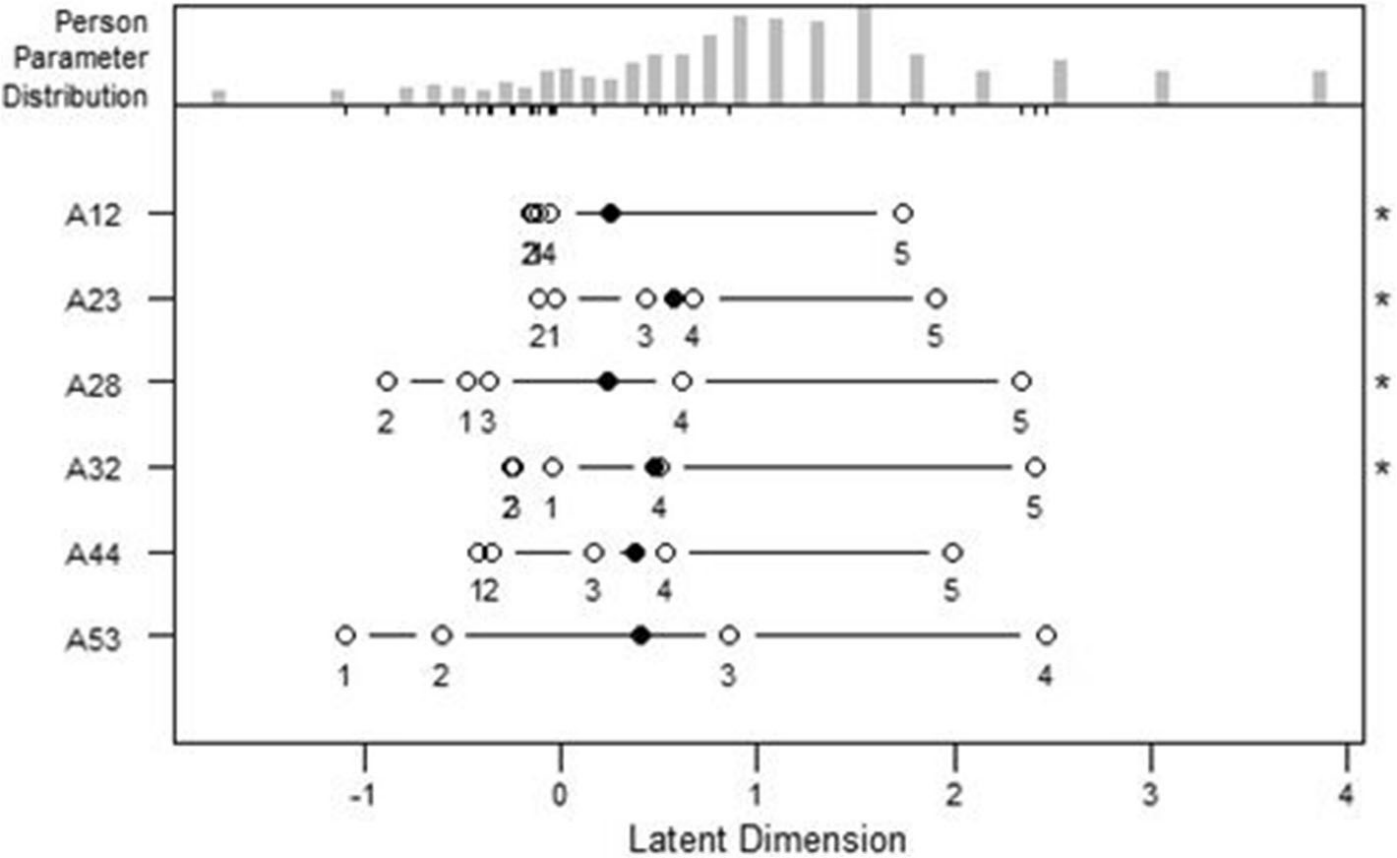
Person-item map for R-PMHI (Development sample)

Four PCRM re-runs were undertaken to select items. An additional file reports the PCRM results and statistics on the items that were removed at the first three runs [See Additional file 1]. In summary, 32 ill-fitting items were deleted in the first run, followed by re-runs with 15, 10 and 6 items. After each run, item content and interpretability of the solution was assessed. The fourth run of PCRM on the six items generated estimates which fit two of the three set criteria for item selection (Table 3). However, four items had the standardized infit and outfit statistics over the set threshold (− 2 to 2). Upon reviewing the content of the six items and conducting a further PCRM run which provided a 2-item solution (results not presented), a decision was made to accept the 6-item solution and stop further item selection. Table 3 and Fig. 1 present the PCRM fit statistics and person-item map for the 6-item R-PMHI in the development sample. Two items were identified as having DIF (Table 4). The item “How often in the last 2 weeks have you felt…Calm” showed DIF by age group, while the item “I make friends easily” had DIF by gender and ethnicity (Chinese vs Indian). The internal consistency reliability was high (Cronbach’s alpha of 0.764).

**Table 3:** Fit statistics for the R-PMHI (Development sample, *n* = 530)

	Subscale source	*X*^2^	df	*p*-value	Outfit MSQ	Infit MSQ	Outfit t	Infit t
I spend time with people I like	ES	388.291	487	1.000	0.80	0.83	−2.69	−2.40
I make friends easily	IS	384.956	487	1.000	0.79	0.78	−3.23	−3.64
I try to be patient with others	IS	428.949	487	0.972	0.88	0.88	−1.77	−1.75
I am willing to share my time with others	IS	374.962	487	1.000	0.77	0.74	−3.39	−4.04
I have freedom to make choices that concern my future	PGA	412.898	487	0.994	0.85	0.88	−2.29	−1.92
How often in the last 2 weeks have you felt ..Calm	GA	508.794	487	0.239	1.04	1.00	0.68	−0.05

**Table 4:** Significant DIF in R-PMHI items by gender, age and ethnicity group comparisons (Development sample, *n* = 530)

		Overall			Slope			Intercept		
	Item	Total *X*^2^	df	P value	*X*^2^_a_	df	P value	*X*^2^_c|a_ (slope)	df	*P* value
Younger (21-39y) vs. older (40-65y)	How often in the last 2 weeks have you felt...Calm	14.8	5	0.011	6.1	1	0.014	8.7	4	0.070
Men vs Women	I make friends easily	15.7	6	0.016	0.8	1	0.374	14.9	5	0.011
Indian vs. Chinese	I make friends easily	13.1	6	0.042	6.5	1	0.011	6.6	5	0.251

**Fig. 2:**
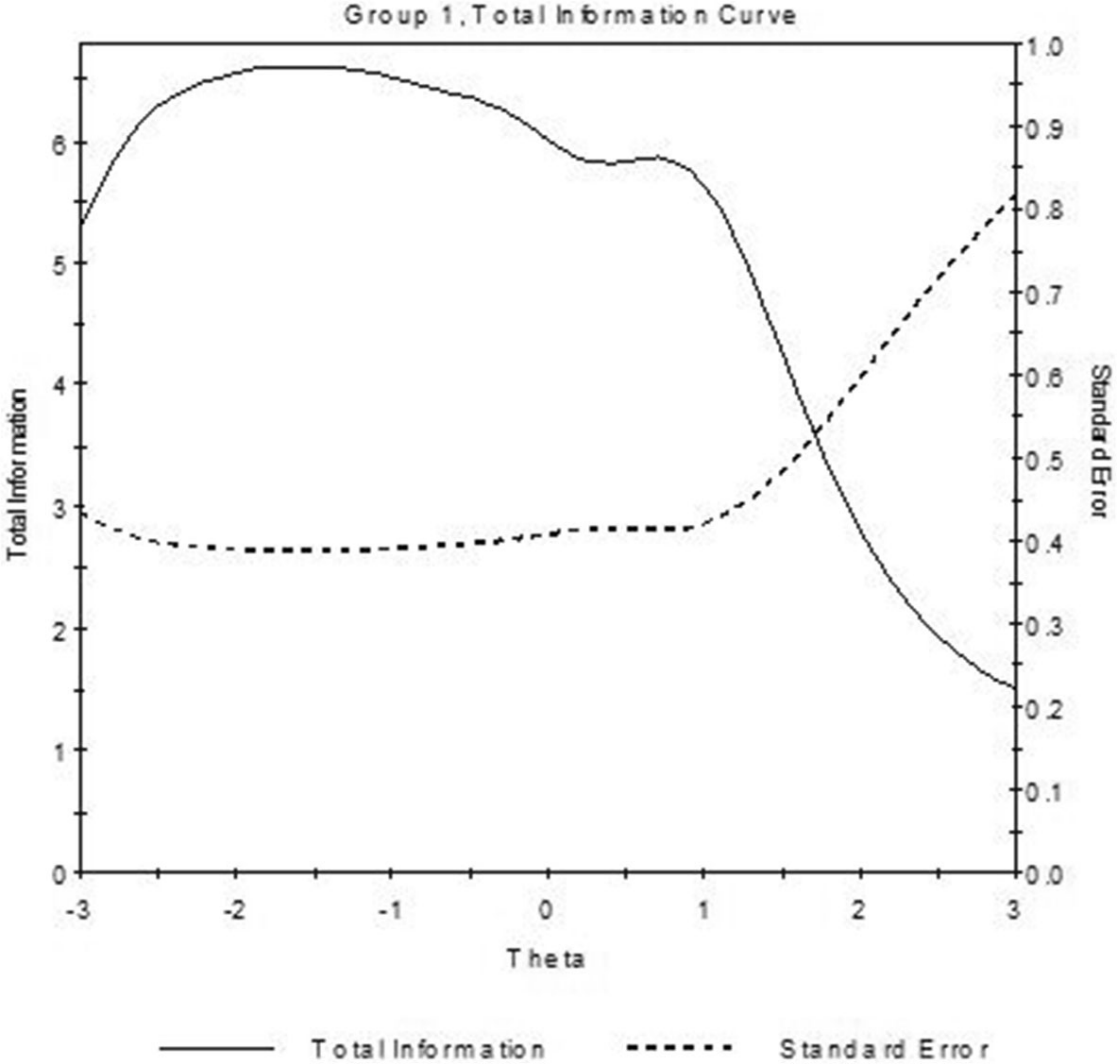
Total information functions curve for R-PMHI using Graded Model Item Parameter Estimates (Internal validation sample)

The CFA in the internal validation sample confirmed the unidimensional structure of the R-PMHI (RMSEA = 0.075, CFI = 0.985, TLI = 0.974). The standardized loadings of the items ranged from 0.394 to 0.799 (Table 5). The item discrimination ranged from 0.24 to 0.57 for scale. The item difficulty estimates for the instrument ranged from − 1.10 to 2.47 (Table 6). Figure 2 displays the TIF curve for the R-PMHI. TIF for R-PMHI had wide width with peak around − 1.8 on its underlying construct axis, which suggests that this scale provides higher precision at the lower end of the continuum (theta < 1).

**Table 5:** Internal and external construct validity by confirmatory factor analysis

	Internal validation sample (n-520)	External validation sample (***N*** = 1925)							
		Overall	Age		Gender		Ethnicity		
			< 40	= > 40	Women	Men	Chinese	Malay	Indian
I spend time with people I like	0.682	0.699	0.707	0.713	0.72	0.677	0.693	0.665	0.726
I make friends easily	0.697	0.726	0.697	0.754	0.713	0.733	0.738	0.683	0.671
I try to be patient with others	0.608	0.702	0.57	0.808	0.741	0.665	0.688	0.715	0.755
I am willing to share my time with others	0.799	0.845	0.839	0.852	0.839	0.85	0.858	0.811	0.854
I have freedom to make choices that concern my future	0.645	0.735	0.669	0.788	0.74	0.73	0.729	0.779	0.722
How often in the last 2 weeks have you felt ..Calm	0.394	0.447	0.444	0.437	0.493	0.41	0.418	0.539	0.441
*X*^2^	35.161	53.728	64.603	36.693	61.928	24.205	69.169	9.461	19.468
df	9	9	9	9	9	9	9	9	9
RMSEA	0.075	0.051	0.082	0.055	0.077	0.043	0.076	0.013	0.056
CFI	0.985	0.992	0.977	0.993	0.980	0.995	0.984	1	0.991
TLI	0.974	0.987	0.987	0.987	0.987	0.987	0.987	0.987	0.987

*df* Degrees of freedom, *RMSEA* Root mean square error of approximation, *CFI* Comparative fit index, *TLI* Tucker-Lewis index

**Table 6:** Item parameter estimates (discriminant and difficulty) (Internal validation sample, *n* = 520)

	a	b1	b2	b3	b4	b5
I spend time with people I like	0.26	−0.11	−0.15	−0.14	−0.06	1.73
I make friends easily	0.57	−0.03	−0.12	0.43	0.68	1.91
I try to be patient with others	0.24	−0.47	−0.89	−0.37	0.61	2.35
I am willing to share my time with others	0.48	−0.04	−0.26	− 0.24	0.50	2.42
I have freedom to make choices that concern my future	0.39	−0.42	−0.36	0.17	0.53	2.00
How often in the last 2 weeks have you felt ..Calm	0.41	−1.10	−0.61	0.86	2.47	**–**

### External validation

The socio-demographic characteristics of the participants in the external validation sample are presented in Table 2. The mean age of the participants was 40.1 years with majority being Chinese (71.1%) followed by Malays (14.1%), Indians (11.0%), and other ethnicities (3.9%).

The unidimensional structure of the R-PMHI was confirmed in the external sample with GOF indices fulfilling the set criteria (RMSEA = 0.051, CFI = 0.992, TLI = 0.987) with high standardized item loadings ranging from 0.447 to 0.845 (Table 5). Its structure also remained strong and met GOF indices when tested by age, gender and ethnic groups (Table 5). The R-PMHI fulfilled concurrent validity criteria showing a significant positive mild correlation with EQ-5D-5 L index score (Spearman’s coefficient of 0.187, *p* < 0.01) and inverse correlation with K6 distress score (Spearman’s coefficient of − 0.316, *p* < 0.01). The internal consistency reliability was high with a Cronbach’s alpha of 0.806. The R-PMHI demonstrated high agreement with the 47-item version with ICC of 0.872 (95% CI: 0.861 to 0.882).

**Fig. 3:**
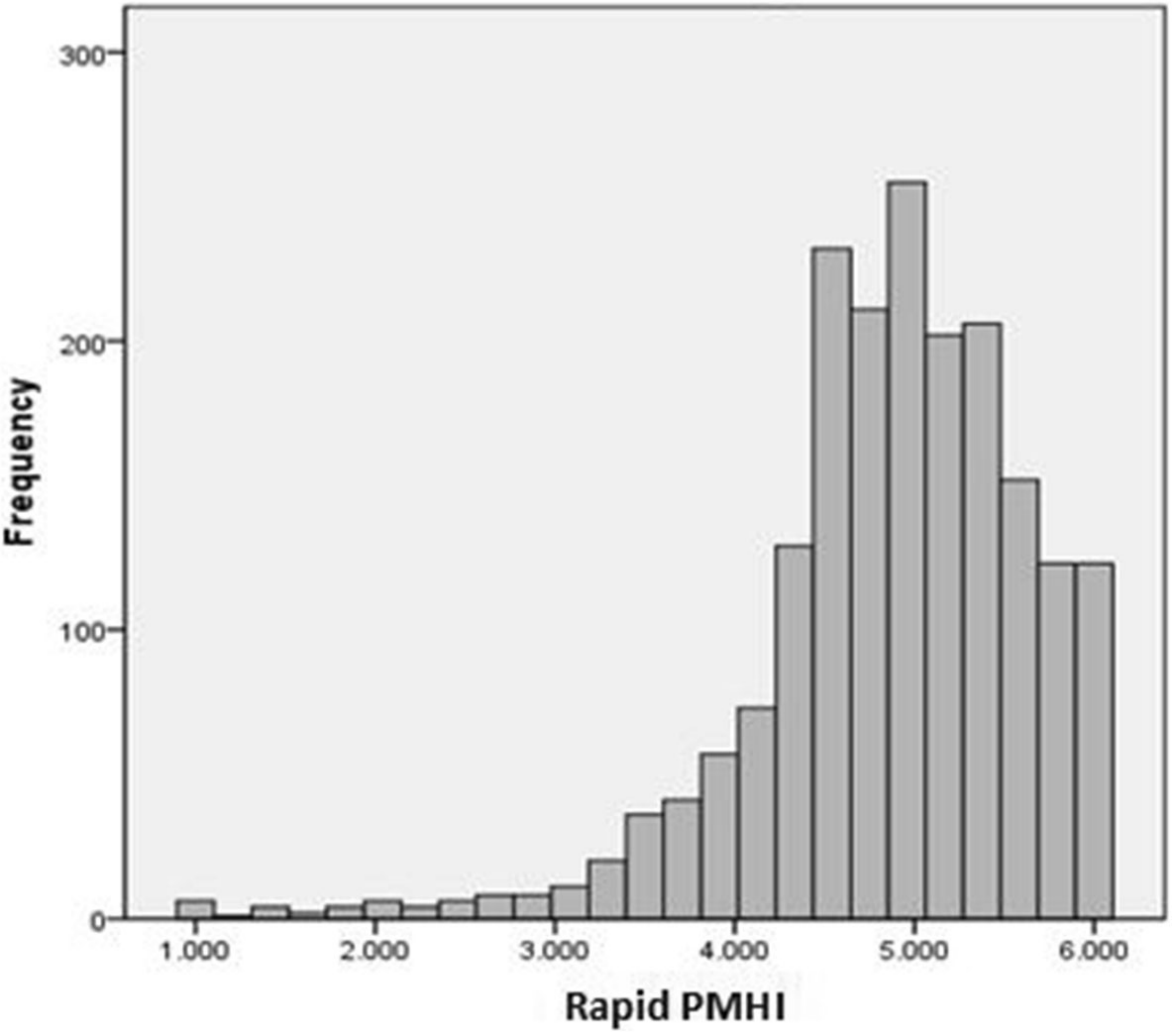
Rapid PMH score distribution in the external validation sample

Weight adjusted mean (SE, 95% CI) R-PMHI score in the population was 4.86 (0.2, 4.82–4.90, Fig. 3). General linear model (GLM) showed ethnicity to be associated with R-PMHI scores after adjusting for the effect of age, gender, marital status and education (Table 7). The weighted floor and ceiling effects for the R-PMHI were 0.3% (unweighted: 0.3%) and 6.0% (unweighted: 6.4%).

**Table 7:** R-PMHI score and socio-demographic correlates in the external validation sample (*n* = 1925)

	Weighted means				GLM on weighted data				
	Mean	SE	95% CI		Beta	SE	95% CI		
			Lower	Upper			Lower	Upper	Sig.
**Age group**									
18–39 years	4.87	0.06	4.75	4.99	−0.061	0.044	−0.147	0.026	0.168
40 y and above	4.93	0.06	4.82	5.04	Ref				
**Gender**									
Men	4.89	0.06	4.77	5.00	−0.027	0.043	−0.111	0.056	0.522
Women	4.92	0.06	4.80	5.03	Ref				
**Ethnicity**									
Malay	4.97	0.06	4.84	5.09	0.191	0.058	0.077	0.305	**0.001**
Indian	4.86	0.07	4.72	5.01	0.088	0.059	−0.027	0.204	0.133
Other	5.00	0.11	4.80	5.21	0.23	0.099	0.035	0.425	**0.021**
Chinese	4.77	0.05	4.67	4.88	Ref	.	.	.	.
**Marital status**									
Never married	4.92	0.06	4.81	5.04	−0.024	0.046	−0.114	0.066	0.601
Divorced/ Separated/ Widowed	4.84	0.09	4.66	5.01	−0.111	0.082	−0.272	0.049	0.174
Married	4.95	0.05	4.84	5.05	Ref				
**Education level**									
Some/Primary	4.86	0.17	4.52	5.20	−0.05	0.177	−0.396	0.296	0.778
Secondary to Junior College	4.92	0.04	4.83	5.00	0.005	0.051	−0.096	0.106	0.923
Vocational / Diploma	4.92	0.05	4.81	5.02	0.005	0.052	−0.097	0.107	0.925
University and above	4.91	0.05	4.82	5.00	Ref				

## Discussion

This study used a Rasch model approach to develop a short unidimensional measure from the 47-item PMHI that can be used to efficiently assess the level of positive mental health among the adult multi-ethnic population in Singapore. The new measure not only showed high agreement with the original measure but it was also valid and reliable, contained acceptable levels of DIF and produced significant correlations in the expected directions with convergent and discriminant measures.

Rasch analysis has been extensively applied in instrument development and improvement in order to ensure that they provide unidimensional measurement of a construct [22]. It is a psychometric technique that increases the precision with which researchers construct instruments taking into account respondents’ performances. The technique provides several advantages in the assessment of health outcomes that are not directly measurable by offering a framework for item selection and the benefit of allowing comparisons across diseases and subgroups [28]. A number of outcome measures including the Short WEMWBS [8], Patient-Reported Outcomes Measurement Information System (PROMIS) [29], EUROHIS-QOL 8-item index, a short version of the 26-item WHO Quality of Life questionnaire (WHOQOL-BREF) [30] and the Multiple Sclerosis Quality of Life (MSQOL)-54 [31] were developed using Rasch analyses. Such patient reported and preference based outcome measures usually have ordinal scales and are prone to high floor or ceiling effects [32]; Rasch analysis has been particularly useful in reducing these. The R-PMHI had very low floor effect (0.3%) and a ceiling effect of 6%. This is high compared with 0.94% ceiling effect seen for total PMH measured by the original measure but much lower than the 10–14% ranges seen for its six subscales [12]. It is possible that items with higher ceiling effect were retained in the 6-item R-PMHI despite the Rasch framework. Since ceiling effect can reduce scale responsiveness or its capacity to detect changes over time [29], it is important to assess this for the R-PMHI in prospective and experimental studies.

Item response theory models (IRT) systematically evaluate item bias using DIF which refers to the situation where members from different groups (e.g., age, gender) who have the same level of positive mental health have a different probability of selecting a certain response to a particular item. IRT-DIF analysis on R-PMHI identified two items with significant DIF (Table 4). The original item selection and development of the 47-item PMHI were based on IRT analyses whereby items with significant IRT-DIF were removed from the item pool [9]. None of the six items retained in the R-PMHI had demonstrated DIF in the general population previously. However, a subsequent investigation among mental health service users had identified one of these items, ‘How often in the last 2 weeks have you felt ..Calm’ to have DIF by age [11]. Similar result was obtained in the current analysis. In addition, the item ‘I make friends easily’ exhibited DIF by gender and ethnicity which was not observed in the earlier studies. The impact of DIF observed in the R-PMHI should be considered while comparing these groups. However, a study in the Singapore general population found that although 20 of the SF-36 questionnaire items had DIF by the history of chronic conditions, its impact on the assessment of health related quality of life was minimal [33]. Scott and colleagues [34] also caution against employing DIF analysis on its own and highlight the importance of assessing other statistical and psychometric results “when deciding whether a particular DIF effect is of sufficient practical importance to require modification of an item or scale”.

The R-PMHI also had four items with infit and outfit statistics over the threshold (− 2 to 2, Table 3). The presence of under fitting items in instruments can potentially impact its validity, whereas overfitting items tend to overestimate differences in raw scores [35]. The former can lead to under-detection of health problems (e.g. false negatives on screening measures), while the latter interferes in comparisons within and between individuals. Although the clinical impact of erroneously removing misfitting items has not been investigated, research indicates that retaining misfitting items has little or no impact on the efficacy of measures [36] and therefore, inclusion of these items in the R-PMHI is unlikely to impact its application. Moreover, while threshold scores below − 2 suggest statistically significant over or under fitting for four items, their mean squares were higher than 0.70 (ranging from 0.74–0.88), suggesting that the actual size of the misfit is not large enough to be concerning.

Given the satisfactory fit to the Rasch model for the 6-item R-PMHI, confirmation of its unidimensionality and the validity of the measure were established in two samples - an internal random split-half sample embedded within the data and an external dataset (Table 5). The R-PMHI also demonstrated high internal consistency reliability in these samples (Cronbach’s alpha of 0.764 and 0.806, respectively). The original PMHI has consistently generated Cronbach’s alphas around 0.9 in previous studies [9, 11, 17]. Although Cronbach’s alphas within 0.70–0.95 indicate superior reliability, high values could indicate item or subscale redundancy [37]. In that, R-PMHI could serve useful by managing the redundancy in the original measure and increasing efficiency of measurements while being psychometrically sound.

The study also estimated preliminary positive mental health values as established by the R-PMHI and explored socio-demographic correlates of positive mental health using the measure. The 47-item PMHI generated values in the range of 3.93 to 4.61 for total positive mental health among populations comprising mental health service users [38] and the general population [12]. The R-PMHI score of 4.86 seen among the general population in this study is much higher than the earlier estimates. While it is possible that participants in the general population sample had good overall mental health, it is also likely that the R-PMHI includes items with higher ceiling effects resulting in a higher composite score. These should be considered while interpreting and comparing R-PMHI scores. Future development of the R-PMHI should involve studies in clinical or vulnerable populations and possible expansion of the 6-point Likert scale to 7 to 10 response options which has been shown to reduce ceiling and floor effects and provide better normalization in the data [39].

R-PMHI scores were associated with ethnicity in this study (Table 7). This finding is consistent with earlier studies [13, 15, 38]. However, R-PMHI scores did not show a significant relationship with marital status as seen in all previous studies using the PMHI where those who were married had significantly higher total PMH as compared to those who were never married and/or were divorced /separated/ widowed [12, 13, 38]. While similar findings were observed with the SWEMWBS in UK [40], the association of marital status with mental well-being components has not been consistent [41, 42]. This highlights the importance of evaluating the living arrangement such as co-habiting over just being married as well as quality of marital life to be considered while investigating this association. This was, however, not captured in our data and remains a future research goal while understanding the R-PMHI score and its correlates.

A challenge, as with any unidimensional scale, is that a single score reflects all the components of positive mental health [43]. Thus, unidimensional rating scales provide relatively less information concerning the comprehensive nature of a construct. Positive mental health being a multidimensional construct ideally requires a multidimensional measure for its assessment. However, the length of the 47-item PMHI poses several challenges for its routine clinical use. It is crucial to evaluate if any construct properties were lost during item reduction. Given that the current short measure did not comprise any items belonging to general coping and spirituality domains (Table 3), it will be of value to evaluate the effect of domain exclusion in future studies.

The six items that remained after the item-reduction partly represent the construct of ‘emotional intelligence’ [44] which relates to understanding one’s emotions as well as others, and believed to comprise one or more of five components - ‘self-awareness, self-regulation, motivation, empathy and social skills’ [45]. Emotional intelligence has been linked to productivity, happiness and psychological well-being and forms an integral part of positive psychology [46, 47]. There are clearly several overlaps and correlations between positive mental health or well-being and emotional intelligence – both can be acquired, are used in targeted interventions in psychotherapy, have shown links with health behaviors such as exercise and linked to self-efficacy, functioning and reduction in depressive symptoms and suicidality [47, 48]. The difference probably lies in their application to mental health promotion; while emotional intelligence has been largely used in reference to students, working adults and specifically healthcare professionals, ‘positive mental health and well-being’ are often applied to population level health promotion. Future research on the R-PMHI would benefit by understanding in what way these constructs differ from or contribute to one another.

### Limitations

Some limitations of this work should be considered while interpreting the results. Although the pooled samples used for this study were large, represented varied settings and included participants from a wide range of socio-cultural backgrounds, majority were recruited via convenience rather than probabilistic sampling. The generalizability of these findings thus warrants further investigation. The development and validation studies were also restricted in the number of variables and measures that were used to assess concurrent validity. Further research will be needed to examine the validity of the R-PMHI in relation to domain-specific positive mental health measures that were employed in the development of the original PMHI. This could provide important information on the construct properties of the short tool vis-a-vis deletion of items belonging to specific subscales. Additionally, the R-PMHI development was guided by a statistical construct and psychometric analyses, where data from previous studies were analyzed without administering the 6-item scale to a new study population. Hence, further investigations and external validation of the measure should be considered. Putting these limitations aside and the need for further research that accompany the development of any new measure, the development and validation of the R-PMHI is adequate whereby it can be offered to adult Asian populations as a brief measure that is reliable, valid, and efficient in the assessment of positive mental health.

## Conclusion

In conclusion, the 6-item R-PMHI has sound psychometric properties which make it useful for studying positive mental health in varied settings. As a validated, short and easily administrable measure, it can be routinely used in large surveys and among clinical populations. Further testing of the scale in diverse samples is needed to further determine its validity, responsiveness and test-retest reliability. While the initial evidence of reliability and validity of the scale is encouraging, future studies should investigate whether the scale addresses the multidimensional positive mental health construct adequately.

## Supplementary information

**Additional file 1.** PCRM run statistics for PMHI. Partial Credit Rasch Model (PCRM) statistics on the items that were removed at the first three analyses runs.

## Data Availability

The datasets generated and/or analysed during the current study are not publicly available due to funding restrictions but are available from the corresponding author on reasonable request.

## Abbreviations

CAPI: Computer-assisted personal interviewing
CFA: Confirmatory factor analysis
CFI: Comparative fit index
DIF: Differential item functioning
EQ-5D-5 L: European Quality of Life-5 Dimensions 5 levels
GOF: Goodness-of-fit
ICC: Intra-class correlation coefficient
IRT-GRM: Item response theory graded response model
K6: Kessler 6 Psychological Distress scale
PAPI: Pen-and-paper personal interviews
PCRM: Partial Credit Rasch Model
PMH: Positive mental health
PMHI: Positive Mental Health Instrument
RMSEA: Root mean square error of approximation
R-PMHI: Rapid Positive Mental Health Instrument
SF-36: Short Form-36
SH-2: Singapore Health-2 study
TIF: Test information function
TLI: Tucker-Lewis index
WEMWBS: Warwick-Edinburgh Mental Well-Being Scale
WHO: World Health Organization
WLSMV: Weighted least squares with mean and variance
