# Supplementary material for: Development and validation of the Rapid Positive Mental Health Instrument (R-PMHI) for measuring mental health outcomes in the population
Source: BMC Public Health. 2020 Apr 10;20:471. doi: 10.1186/s12889-020-08569-w (PMC7146878; doi:10.1186/s12889-020-08569-w)
Supplement: Supplementary file 1 — Additional file 1. PCRM run statistics for PMHI. Partial Credit Rasch Model (PCRM) statistics on the items that were removed at the first three analyses runs. [file 12889_2020_8569_MOESM1_ESM.docx]

**Additional File 1: PCRM statistics for PMHI**

| **No** | **Items** | **Subscale** | **Chisq** | **df** | **p-value** | **Outfit MSQ** | **Infit MSQ** | **Outfit t** | **Infit t** | **Decision** |
| --- | --- | --- | --- | --- | --- | --- | --- | --- | --- | --- |
| **Run 1** |  |  |  |  |  |  |  |  |  |  |
| 1 | A1 | GC | 464.37 | 501 | 0.878 | 0.93 | 0.89 | -1.13 | -1.82 | Select |
| 2 | A2 | GC | 465.14 | 501 | 0.873 | 0.93 | 0.94 | -1.14 | -1.03 | Select |
| 3 | A3 | GC | 400.43 | 501 | 1.000 | 0.80 | 0.81 | **-3.10** | -3.16 |  |
| 4 | A5 | GC | 506.78 | 501 | 0.420 | 1.01 | 1.02 | 0.17 | 0.32 | Select |
| 5 | A6 | GC | 548.84 | 501 | 0.069 | 1.09 | 1.04 | 1.44 | 0.73 | Select |
| 6 | A7 | GC | 555.48 | 501 | **0.046** | 1.11 | 1.08 | 1.54 | 1.17 |  |
| 7 | A9 | GC | 450.94 | 501 | 0.947 | 0.90 | 0.87 | -1.59 | **-2.25** |  |
| 8 | A10 | GC | 780.52 | 501 | **0.000** | 1.56 | 1.27 | 7.45 | 4.34 |  |
| 9 | A11 | GC | 457.20 | 501 | 0.920 | 0.91 | 0.92 | -1.33 | -1.24 | Select |
| 10 | A12 | ES | 521.26 | 501 | 0.257 | 1.04 | 1.01 | 0.49 | 0.18 | Select |
| 11 | A13 | ES | 678.86 | 501 | **0.000** | 1.35 | 1.18 | 4.58 | 2.69 |  |
| 12 | A14 | S | 612.75 | 501 | **0.000** | 1.22 | 1.04 | 2.48 | 0.69 |  |
| 13 | A16 | IS | 417.74 | 501 | 0.997 | 0.83 | 0.76 | **-2.31** | -3.59 |  |
| 14 | A17 | ES | 451.03 | 501 | 0.947 | 0.90 | 0.90 | -1.31 | -1.41 | Select |
| 15 | A19 | ES | 868.60 | 501 | **0.000** | 1.73 | 0.97 | 7.04 | -0.39 |  |
| 16 | A20 | ES | 580.03 | 501 | **0.008** | 1.16 | 1.00 | 2.00 | -0.06 |  |
| 17 | A21 | ES | 508.67 | 501 | 0.397 | 1.01 | 0.92 | 0.21 | -1.37 | Select |
| 18 | A22 | ES | 439.75 | 501 | 0.977 | 0.88 | 0.90 | -1.80 | -1.57 | Select |
| 19 | A23 | IS | 466.28 | 501 | 0.865 | 0.93 | 0.94 | -1.04 | -0.98 | Select |
| 20 | A24 | IS | 412.31 | 501 | 0.998 | 0.82 | 0.86 | **-2.54** | -2.00 |  |
| 21 | A25 | IS | 423.72 | 501 | 0.995 | 0.84 | 0.90 | **-2.27** | -1.47 |  |
| 22 | A27 | IS | 662.04 | 501 | **0.000** | 1.32 | 1.16 | 4.29 | 2.31 |  |
| 23 | A28 | IS | 499.06 | 501 | 0.516 | 0.99 | 0.98 | -0.18 | -0.27 | Select |
| 24 | A30 | IS | 736.96 | 501 | **0.000** | 1.47 | 1.21 | 5.81 | 2.94 |  |
| 25 | A31 | IS | 658.94 | 501 | **0.000** | 1.31 | 1.02 | 3.97 | 0.36 |  |
| 26 | A32 | IS | 477.55 | 501 | 0.768 | 0.95 | 0.94 | -0.65 | -0.87 | Select |
| 27 | A34 | S | 936.48 | 501 | **0.000** | 1.87 | 1.42 | 7.61 | 5.82 |  |
| 28 | A35 | S | 743.16 | 501 | **0.000** | 1.48 | 1.26 | 5.48 | 4.08 |  |
| 29 | A36 | S | 725.34 | 501 | **0.000** | 1.45 | 1.30 | 4.59 | 4.39 |  |
| 30 | A37 | S | 745.73 | 501 | **0.000** | 1.49 | 1.27 | 4.49 | 3.90 |  |
| 31 | A39 | S | 1161.6 | 501 | **0.000** | 2.31 | 1.47 | 12.82 | 6.95 |  |
| 32 | A40 | S | 868.86 | 501 | **0.000** | 1.73 | 1.34 | 7.91 | 5.08 |  |
| 33 | A41 | PGA | 338.88 | 501 | 1.000 | 0.68 | 0.71 | **-5.32** | -4.80 |  |
| 34 | A42 | PGA | 444.52 | 501 | 0.967 | 0.89 | 0.86 | -1.69 | **-2.23** |  |
| 35 | A43 | PGA | 467.03 | 501 | 0.859 | 0.93 | 0.84 | -1.09 | **-2.64** |  |
| 36 | A44 | PGA | 451.44 | 501 | 0.945 | 0.90 | 0.93 | -1.47 | -1.03 | Select |
| 37 | A46 | PGA | 677.32 | 501 | **0.000** | 1.35 | 1.07 | 4.89 | 1.18 |  |
| 38 | A47 | PGA | 431.92 | 501 | 0.988 | 0.86 | 0.85 | -2.00 | **-2.33** |  |
| 39 | A48 | PGA | 362.41 | 501 | 1.000 | 0.72 | 0.78 | **-4.33** | **-3.65** |  |
| 40 | A49 | PGA | 379.54 | 501 | 1.000 | 0.76 | 0.76 | **-3.91** | **-3.86** |  |
| 41 | A50 | PGA | 359.75 | 501 | 1.000 | 0.72 | 0.76 | **-4.59** | **-4.11** |  |
| 42 | A52 | PGA | 366.32 | 501 | 1.000 | 0.73 | 0.79 | **-4.29** | **-3.34** |  |
| 43 | A53 | GA | 489.64 | 501 | 0.633 | 0.98 | 0.94 | -0.37 | -0.94 | Select |
| 44 | A54 | GA | 431.75 | 501 | 0.989 | 0.86 | 0.79 | **-2.29** | -3.64 |  |
| 45 | A55 | GA | 428.09 | 501 | 0.992 | 0.85 | 0.84 | **-2.41** | -2.67 |  |
| 46 | A56 | GA | 445.06 | 501 | 0.965 | 0.89 | 0.88 | -1.90 | **-2.11** |  |
| 47 | A57 | GA | 522.84 | 501 | 0.242 | 1.04 | 0.93 | 0.68 | -1.11 | Select |
|  |  |  |  |  |  |  |  |  |  |  |
| **Run 2** |  |  |  |  |  |  |  |  |  |  |
| 1 | A1 | GC | 412.14 | 494 | 0.997 | 0.833 | 0.837 | **-2.620** | -2.66 |  |
| 2 | A2 | GC | 465.37 | 494 | 0.818 | 0.940 | 0.964 | -0.920 | -0.560 | Select |
| 3 | A5 | GC | 452.56 | 494 | 0.909 | 0.914 | 0.980 | -1.310 | -0.280 | Select |
| 4 | A6 | GC | 521.91 | 494 | 0.186 | 1.054 | 1.018 | 0.850 | 0.310 | Select |
| 5 | A11 | GC | 460.15 | 494 | 0.860 | 0.930 | 0.969 | -1.030 | -0.460 | Select |
| 6 | A12 | ES | 430.49 | 494 | 0.982 | 0.870 | 0.944 | -1.670 | -0.740 | Select |
| 7 | A17 | ES | 412.13 | 494 | 0.997 | 0.833 | 0.856 | **-2.230** | -2.120 |  |
| 8 | A21 | ES | 489.74 | 494 | 0.546 | 0.989 | 0.869 | -0.120 | **-2.160** |  |
| 9 | A22 | ES | 385.29 | 494 | 1.000 | 0.778 | 0.814 | **-3.320** | -3.080 |  |
| 10 | A23 | IS | 465.10 | 494 | 0.820 | 0.940 | 0.973 | -0.870 | -0.410 | Select |
| 11 | A28 | IS | 540.30 | 494 | 0.073 | 1.092 | 1.067 | 1.290 | 0.940 | Select |
| 12 | A32 | IS | 459.46 | 494 | 0.865 | 0.928 | 0.914 | -0.980 | -1.220 | Select |
| 13 | A44 | PGA | 457.49 | 494 | 0.879 | 0.924 | 0.971 | -1.090 | -0.410 | Select |
| 14 | A53 | GA | 507.75 | 494 | 0.325 | 1.026 | 0.992 | 0.420 | -0.100 | Select |
| 15 | A57 | GA | 573.67 | 494 | **0.008** | 1.159 | 1.019 | **2.430** | 0.330 |  |
|  |  |  |  |  |  |  |  |  |  |  |
| **Run 3** |  |  |  |  |  |  |  |  |  |  |
| 1 | A2 | GC | 404.90 | 491 | 0.998 | 0.823 | 0.85 | **-2.86** | -2.48 |  |
| 2 | A5 | GC | 378.86 | 491 | 1 | 0.77 | 0.816 | **-3.72** | -3 |  |
| 3 | A6 | GC | 435.08 | 491 | 0.967 | 0.884 | 0.856 | -1.83 | **-2.39** |  |
| 4 | A11 | GC | 412.92 | 491 | 0.996 | 0.839 | 0.88 | **-2.46** | -1.91 |  |
| 5 | A12 | ES | 508.72 | 491 | 0.281 | 1.034 | 0.958 | 0.44 | -0.55 | Select |
| 6 | A23 | IS | 483.45 | 491 | 0.587 | 0.983 | 0.964 | -0.23 | -0.55 | Select |
| 7 | A28 | IS | 463.95 | 491 | 0.805 | 0.943 | 0.941 | -0.8 | -0.82 | Select |
| 8 | A32 | IS | 463.10 | 491 | 0.812 | 0.941 | 0.9 | -0.79 | -1.45 | Select |
| 9 | A44 | PGA | 434.27 | 491 | 0.969 | 0.883 | 0.942 | -1.71 | -0.86 | Select |
| 10 | A53 | GA | 527.34 | 491 | 0.124 | 1.072 | 1.012 | 1.11 | 0.2 | Select |

**Key:**

| GC | General Coping | IS | Interpersonal Skills |
| --- | --- | --- | --- |
| ES | Emotional Support | PGA | Personal Growth and Autonomy |
| S | Spirituality | GA | Global Affect |
